# Enhancing the effectiveness of infectious disease health education for children and adolescents in China: a national multicenter school-based trial

**DOI:** 10.1186/s12889-023-16000-3

**Published:** 2023-06-15

**Authors:** Xinxin Wang, Jieyu Liu, Yu Wu, Binbin Su, Manman Chen, Qi Ma, Tao Ma, Li Chen, Yi Zhang, Yanhui Dong, Yi Song, Jun Ma

**Affiliations:** 1 School of Public Health, Ningxia Medical University, Yinchuan, China; 2 Key Laboratory of Environmental Factors and Chronic Release Control, Yinchuan, China; 3 Institute of Child and Adolescent Health, School of Public Health, National Health Commission Key Laboratory of Reproductive Health, Peking University, Beijing, 100191 China; 4 School of Population Medicine and Public Health, Chinese Academy of Medical Sciences / Peking Union Medical College, Beijing, China

**Keywords:** China, Children and adolescents, Infectious disease, Intervention, Social-ecological model

## Abstract

**Background:**

Infectious diseases pose a significant risk to the health and well-being of children and adolescents, and can even be life-threatening. Thus, our study aimed to explore the effectiveness of health education based on the social-ecological model in improving the knowledge of infectious diseases among this vulnerable population.

**Methods:**

This study was a school-based intervention conducted in seven Chinese provinces in 2013, involving a total of 26,591 children and adolescents in the intervention group and 24,327 in the control group. The intervention group received a comprehensive health intervention based on the social-ecological model (SEM) over six months, which included a supportive environment, health education on infectious diseases, guidance on self-monitoring infectious disease-related behaviors, and other measures. Data on infectious disease-related knowledge and other characteristics were collected through questionnaires. The main outcome measure will be the difference in the effectiveness of health education regarding infectious diseases in children and adolescents between baseline and post-intervention. A mixed-effects regression model was used to calculate the odds ratio (OR) and 95% confidence interval (95% CI) to assess the impact of infectious disease-related interventions on the participants.

**Results:**

We utilized a socioecological model as the foundation for a six-month health education program on infectious diseases targeting children and adolescents in the intervention group. At the individual and community levels, the correct rate of health behavior related to infectious diseases in the intervention group was higher than that in the control group (P < 0.05), the ORs (95% CI) were 0.94 (0.90–0.99) and 0.94 (0.89–0.99), respectively. But the intervention effect was not significant at the interpersonal level. The intervention effect at the organizational level was obvious, with an increase in opportunities for children and adolescents to acquire knowledge of infectious diseases from courses and lectures, teachers, and doctors, (all P < 0.05), with the ORs (95% CI) of 0.92 (0.87–0.97) and 0.86 (0.83–0.94), respectively. However, there was no significant difference between the intervention group and the control group in school infectious disease health education policy.

**Conclusion:**

Enhancing health education regarding infectious diseases is crucial in promoting comprehensive prevention and control measures among children and adolescents. Nevertheless, it remains imperative to reinforce health education on infectious diseases at the interpersonal and policy levels. This holds significant reference value for mitigating childhood infectious diseases in the post-COVID-19 era.

**Supplementary Information:**

The online version contains supplementary material available at 10.1186/s12889-023-16000-3.

## Introduction

Infectious diseases are significant burdens on public health and the economic stability of societies, which have for centuries been the leading cause of death and disability and present growing challenges to health security and human progress. From a worldwide perspective, an estimated 57 million people died each year as a direct result of infectious diseases [1]. Apart from this, emerging infectious diseases present epidemic, even pandemic trends [2], such as Corona Virus Disease 2019 (COVID-19), monkeypox, and Respiratory syncytial virus [3, 4]. In the context of global and national epidemics of infectious diseases, the transition of epidemiological characteristics and the disease spectrum of different infectious diseases brought new impacts on population health. For a long time, infectious diseases threaten children and adolescents’ health and lives [5]. Children and adolescents have historically been particularly susceptible to life-threatening complications from infectious diseases, making them a focus of public health policy in China [6]. Thus, strengthening interventions to address infectious diseases among this population is crucial for tracking trends and developing effective prevention and control policies. However, there is limited research on the efficacy of health education for infectious diseases among Chinese pediatric populations.

Promoting infectious diseases health education among children and adolescents in China is crucial for effectively managing outbreaks and preventing incidence in the community [7, 8]. Increased knowledge of infectious diseases could also lead to improvements in dietary habits and lifestyles [9, 10]. While measures such as clean drinking water, toilet sanitation, and health education have been shown to prevent infectious diseases in schools, research on such measures has been neglected with the reduction of infectious diseases due to improvements in the country’s economic status [11–13]. It was important to recognize the continued importance of such measures in preventing and managing infectious diseases in China. Although health education is a crucial component of health interventions, various internal and environmental factors may impede the improvement of children’s and adolescent’s health [14]. While previous studies have attempted to identify and understand factors contributing to inadequate levels of knowledge about infectious diseases among this population, they have primarily focused on individual-level factors. One intervention study on infectious diseases in the US showed that receiving a brief classroom LD education program based on social learning theory and HBM improved children’s knowledge, attitudes, and self-reported preventive behaviors about Lyme disease [15]. Similarly, a school-based water, sanitation, and hygiene intervention study in the Philippines showed that students’ hygiene behaviors improved significantly in the group that received hygiene education intervention [9]. Participation in health-enhancing behaviors is not merely a matter of individual decisions or intentions but is also influenced by the social and physical environment [16, 17]. To identify the key factors that influence public engagement and understand their relationships, many researchers have advocated for the use of social-ecological modeling as an organizational framework [18, 19]. McLeroy etc. (1988) [19] proposed a social-ecological model (SEM) that groups influence into five levels: individual, interpersonal, organizational, community, and policy levels. This ecological framework overcomes several limitations of individual-based behavioral and psychosocial theories by incorporating a broad range of influences at multiple levels, particularly by adding interpersonal, environmental, and policy factors that interact at different levels and influence specific health behaviors.

This study was a multi-centered, cluster-controlled trial, involving more than 50,000 children and adolescents aged 6–18 from seven provinces in China. Compared with individual or other level intervention studies based on children and adolescents alone, comprehensive intervention studies based on SEM can comprehensively understand the health problems of children and adolescents related to infectious diseases. Given the current devastating incidence of infectious diseases in children and adolescents, improving health education is an important way to control epidemics and outbreaks of infectious diseases [20]. After 6 months of intervention, the intervention effect of infectious disease health education in children and adolescents was observed, which provided a basis for further formulation and optimization of infectious disease prevention and control policies for children and adolescents, especially in the post-epidemic era of COVID-19.

## Materials and methods

### Study design and participants chosen

**Fig. 1:**
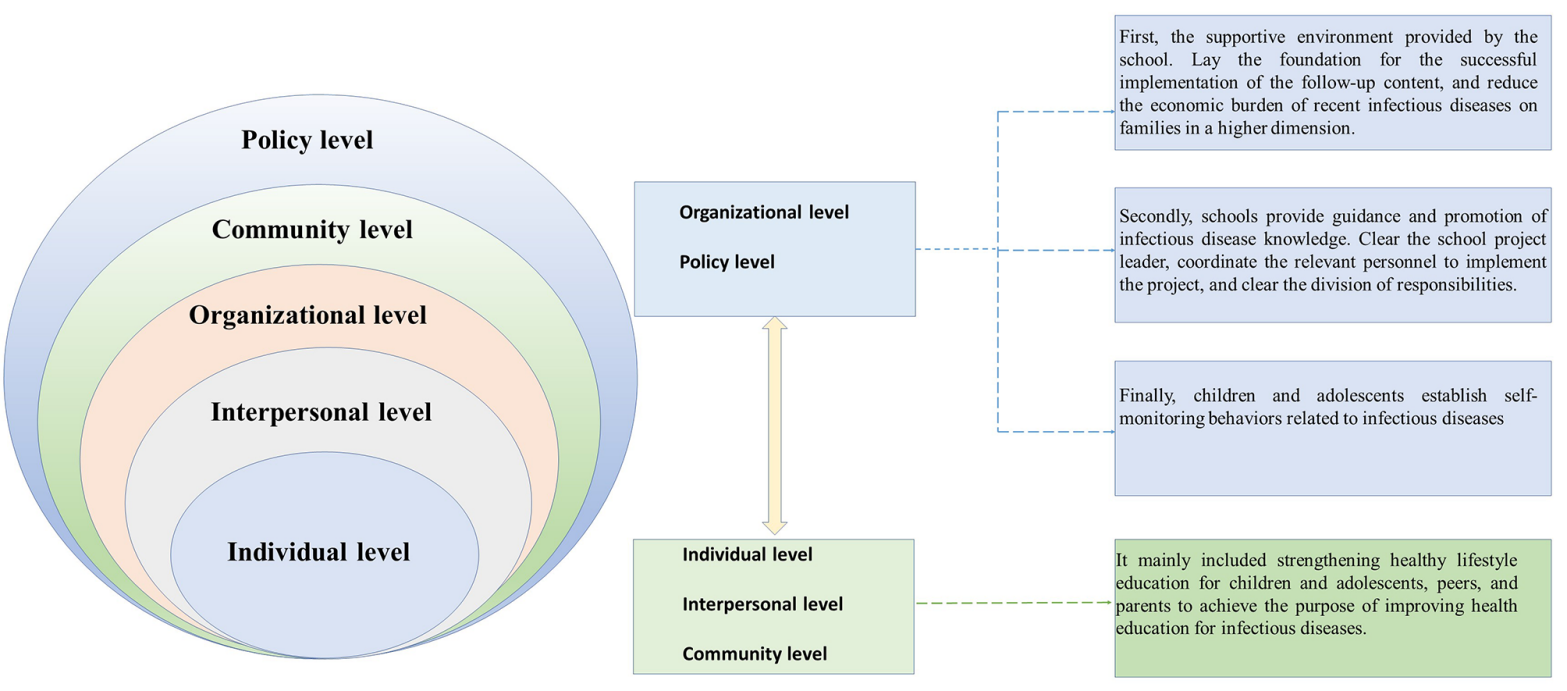
Infectious disease prevention interventions

**Fig. 2:**
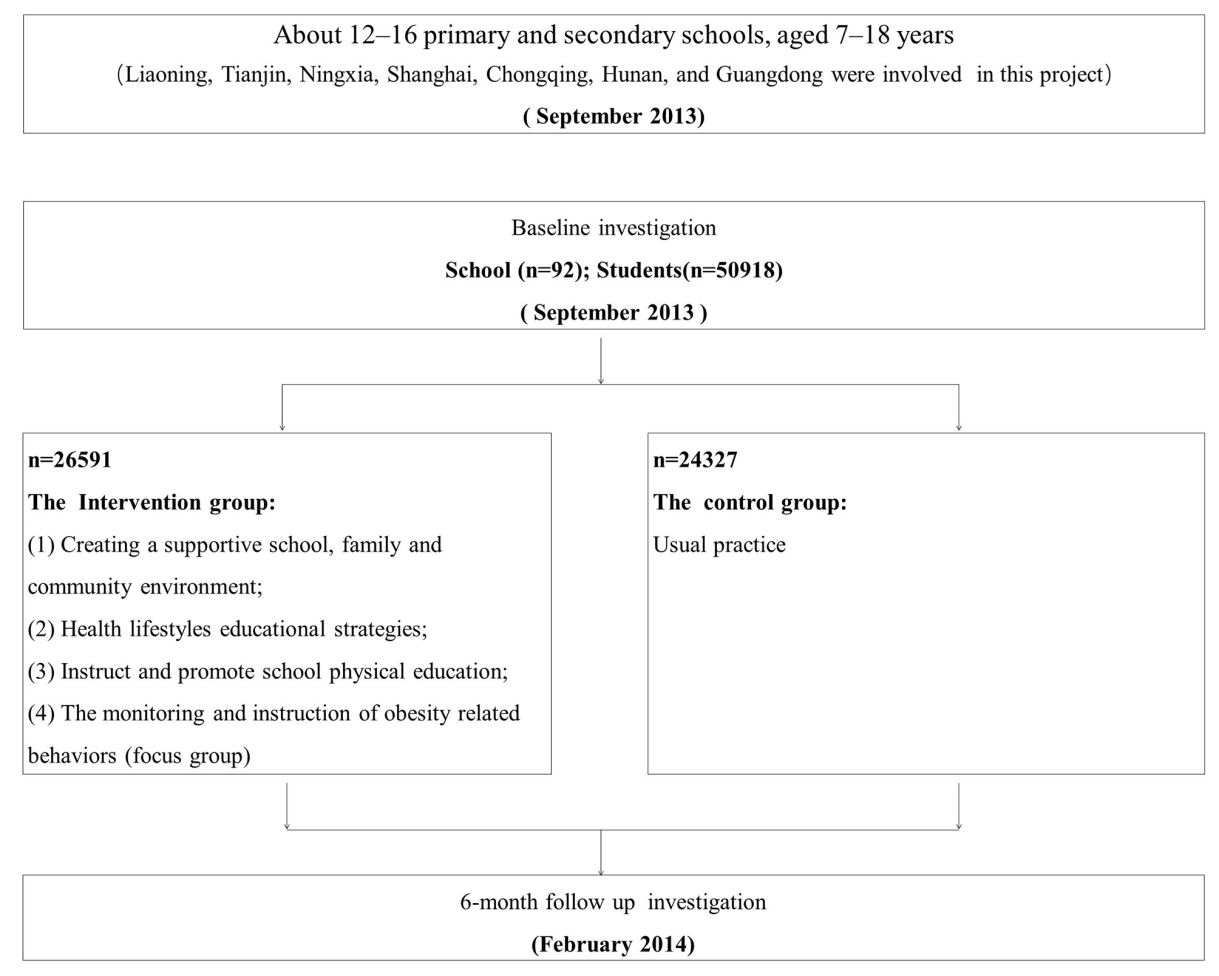
Trial procedures and interventions received by subjects

This national trial with a multi-centered, cluster-controlled trial design involving seven provinces of Liaoning, Tianjin, Ningxia, Shanghai, Chongqing, Hunan, and Guangdong. The project was a 6-month intervention from September 2013 to February 2014. The project observed the impact of interventions on health education for infectious diseases based on SEM through Chinese Children and Adolescents Healthy Lifestyle Intervention (HLI-CCA), as Fig. 1 describes. The standardized and uniform research protocol was applied in all intervention schools in the seven provinces. Figure 2 shows the flow of participants through the trial in each center. First, seven provinces in China were chosen as intervention centers based on their regional and economic status. Second, a representative sample of children and adolescents from each center was obtained using multistage whole-group nonrandom sampling to select schools that met the survey requirements, were willing to participate, and met the inclusion criteria as per the trial protocol. Finally, 12–17 primary and secondary schools were enrolled in each center, totaling 92 schools, which were assigned to either the control or intervention group. In each school, participants were selected from n classes per grade level, with n depending on the average class size, ensuring that no school had fewer than 200 students. The protocol was adjusted slightly to meet the matching criteria for balanced schools in the same stratum in each center and to achieve an equal distribution of participants in the control and intervention groups (i.e., boy/girl = 1:1, primary school/secondary school = 1:1, urban/rural = 1:1, control/intervention = 1:1). Among the original surveyed population of 65,347 participants aged 6–18 years, 14,429 participants were excluded from the present analysis because of missing information on the infectious disease knowledge questionnaire, making the final sample size 50,918. All the children and adolescents from selected classes were invited to participate. The study has been approved by the ethical committee of Peking University (number: IRB0000105213034). In both data collections, children and adolescents and their parents or legal guardians obtained written informed consent.

### Intervention framework and package

The multifaceted intervention was developed based on a socio-ecological model, with a duration of 6 months. Briefly, the intervention consisted of five areas: one directly targeting the individual, interpersonal, and community levels, (mainly health education activities for children and adolescents, peers, and parents), and three targeting the organizational and policy levels (e.g. guidance to principals/teachers and implementation of school policies). A more detailed description of the implemented interventions is presented in Fig. 1. The schools in the control group will not receive any interventions during the course of the project. Additionally, project-related intervention materials will be provided to the control group schools after the entire project is completed.

At the individual, interpersonal, and community levels, it mainly included strengthening healthy lifestyle education for children and adolescents, peers, and parents to achieve the purpose of improving health education for infectious diseases. The specific measures are as follows: (a) Health education (lectures, theme class meetings) for children and adolescents and their parents once a week, 30 min each time. (b) Health knowledge and skills were taught through classroom lectures, visits, face-to-face coaching, and group activities. (c) Schools and families provided necessary health facilities. (d) Teachers provided health education for children and adolescents, peers and parents, train small guardians to prevent diseases and enhance students’ sense of ownership. (e) Volunteer activities for children and adolescents, peers, and parents were established. (f) Encouragement and support for children, adolescents, and parents to disseminate health knowledge to the community.

At the organizational and policy levels: First, the supportive environment was provided by the school. The specific measures are as follows: (a) Provide information on the prevention of infectious diseases (through posters, radio, and websites) and create a healthy campus. (b) Create a healthy school sanitation environment: provide boiled water; hand hygiene products including soap or hand sanitizer. (c) Standardize school health education curriculum according to national standards. Secondly, schools guide infectious disease knowledge. The specific measures were as follows: (a) Design the health education curriculum. (b) Hold health education activities according to health activity prescriptions. (c) Formulate and implement relevant policies to guide health education for children and adolescents. Finally, children and adolescents establish self-monitoring behaviors related to infectious diseases, with the specific measures as follows: (a) The project team regularly monitors the health status of children and adolescents. (b) School supervisor provides regular feedback.

### Data collection and measurement

The parents’ self-administered questionnaires included information such as household monthly income, parents’ occupation, and parents’ educational level. The number of children in the family was summarized as “1” and “≥1”. The educational attainment of parents was surveyed and categorized as “Junior high school and below”, “high school and Junior College” and “College and above”. Parents’ occupations were surveyed and grouped into “administrators and clerks”, “professional technicians”, “business and services” and “others”. In addition, the living area was divided into rural and urban areas, the monthly household income was defined by the sum of all household members’ monthly income (in CNY), and then classified as “<2,000”, “2,000–5,000”, “5,000–8,000”. In the pre-experiment, we found that parents were extremely aware of their children’s behaviors and could understand and question well, so questionnaires for children in grades 1–3 were reported by parents. Children and adolescents in grade 4 and above completed the questionnaire themselves under the guidance of teachers. The questionnaire mainly collects the behavioral lifestyles of children and adolescents related to infectious diseases. According to the five parts of the social-ecological model (SEM), each part collects two questions.

### Investigation process

A national multi-center cluster intervention trial was used. Selected children and adolescents aged 7–18 from various schools were invited to participate in the survey. After a school was confirmed to be eligible and both parent’s and children’s written informed consent had been obtained, it was randomly assigned to intervention or control groups. Only schools in the intervention group were given the intervention throughout the study period, and the duration of the intervention was 6 months. The control group schools received no intervention. After the completion of the entire project, the schools in the control group were also receive project-related intervention materials. The questionnaires were collected twice, at baseline and 6 months after the intervention.

### Quality control

Before the start of the project, intervention school physicians and health education teachers underwent training by professional project members. Specialized personnel were assigned to supervise the intervention schools throughout the program. The project team developed and provided all advocacy and educational materials. Project managers conducted two supervisory visits to the intervention schools during the intervention period. To mitigate the loss of follow-up, incentives were provided, including intervention materials for completing the pilot, education department credits for the school, and financial incentives for health education teachers and school physicians.

### Statistical analysis

Baseline characteristics were described as the mean (SD) for continuous variables or the number (percentage) for categorical variables. The analysis of the mixed-effects regression models was used to evaluate the effect of the intervention on health education related issues after adjusting for age, sex, provinces, urban/rural areas, and the baseline disequilibrium for multiple variables. OR values < 1 meant an increased effect in the variables after intervention with significant p-values, while OR value > 1 meant an effective declined effect. Furthermore, a series of stratified analyses based on residence areas and age (three age groups 6–11, 12–14, and 15–18 years) were performed to determine the effective or sensitive subgroups of the intervention. Statistical analyses were conducted using SPSS 25.0 (IBM SPSS Statistics 25.0, USA) and Stata version 14.0 (Stata Corp). Statistical significance was defined using a two-sided test with *P* values of 0.05.

## Result

### General characteristics

A total of 26,591 children and adolescents in the intervention group and 24,327 in the control groups entered the final analysis. Table 1 shows the characteristics of the study population. The mean age was 10.7 ± 3.3 years, and 10.9 ± 3.3 years in the intervention group and control group, respectively. Only-child families accounted for 69.1% and 67.5% of the intervention and control groups, respectively (*P < 0.001*). In terms of education, 31.97% and 25.18% of fathers in the intervention and control groups had received college and above education, while 28.81% and 23.23% of mothers had received high school education (*P < 0.001*). Regarding occupation, 3.8% of the children’s fathers were administrators and clerks, 13.1% were professional technicians, and 35.4% were business and service workers (*P < 0.001*). A similar trend was noted for the mothers’ occupation. At the individual, interpersonal and community levels. Supplementary Table 1 presents an overview of the key characteristics of the study population, categorized by school levels.

### At the individual, interpersonal and community levels

As shown in Table 2, following an average of six months of health education interventions children and adolescents in the intervention group exhibited higher levels of infectious disease-related health behaviors at the individual level in comparison to the control group (all *P < 0.05*). The odds ratios (95% CI) were 0.94 (0.90–0.99) and 0.94 (0.89–0.99). Table S4 shows that the lower the educational level of the parents, the better the intervention effect. However, the intervention effect was not significant at the interpersonal level. Similar outcomes were observed across different regions and age groups, as outlined in Table S2 and Table S3. Notably, at the community level, although boys in the intervention group did not display healthier behaviors than girls in public places, their behaviors exhibited a positive change with an odds ratio of 0.93 (95% CI = 0.87-1.00, *P < 0.05*). At the organizational and policy levels.

### At the organizational and policy levels

Table 2 demonstrates a significant positive impact of the intervention at the organizational level. The implementation of intervention measures led to a substantial increase in opportunities for children and adolescents to acquire knowledge about infectious diseases from courses, lectures, and teachers (*P < 0.05*). The odds ratios (95% confidence intervals) were 0.92 (0.87–0.97) and 0.86 (0.83–0.94), respectively. These trends were consistent across various regions and age groups (*P < 0.05*). The subgroup analysis further revealed a more significant increase in access to health knowledge in urban areas compared to rural areas (refer to Table S2). Similarly, both the 9–11 and 12–14 age groups experienced a similar improvement in access to health knowledge following the corresponding intervention measures (refer to Table S3). However, there was no significant difference observed between the intervention and control groups regarding school infectious disease health education policy, and this result remained consistent across various regions and age groups, as outlined in Table S2 and Table S3.

**Table 1:** Baseline characteristics between intervention and control groups of the study population

Characteristics	Intervention group	Control group	t/χ^2^	*p-*value
N	26,591	24,327		
Boys (%)	50.76	50.77	-0.020	0.984
Age(years)	10. 7(3.29)	10.92 (3.28)	-8.513	< 0.001
City(%)	16,022(60.25)	14,907(61.28)	-2.364	0.018
Primary caregivers, n (%)				
Parents	23,037(89.23)	21,234(89.53)	-1.064	0.287
Socio-demographics, n (%)				
Only child	18,373(69.09)	16,430(67.54)	3.773	< 0.001
Father’s educational level				
Junior high school and below	9767(40.48)	10,457(47.14)	-17.312	< 0.001
High School and Junior College	6646(27.55)	6141(27.68)		
College and above	7712(31.97)	5585(25.18)		
Mother’s educational level				
Junior high school and below	10,697(44.39)	11,360(51.36)	-16.245	< 0.001
High School and Junior College	6457(26.80)	5623(25.42)		
College and above	6943(28.81)	5138(23.23)		
Father occupation				
Administrator and clerk	1614(8.33)	1118(6.38)	9.980	< 0.001
Professional and technical	2958(15.27)	2475(14.13)		
Commerce and services	6673(34.44)	5762(32.90)		
Other	8128(41.96)	8158(46.58)		
Mather occupation				
Administrator and clerk	772(3.75)	485(2.59)	9.600	< 0.001
Professional and technical	2692(13.08)	2157(11.50)		
Commerce and services	7275(35.35)	6412(34.19)		
Other	9842(47.82)	9702(51.73)		
Monthly household income (RMB)				
< 2000	2031(16.83)	2127(18.16)	-2.752	0.006
2,000–5,000	5882(48.74)	5690(48.57)		
5,000–8,000	4156(34.44)	3898(33.27)		

Note: Other occupations of father and mother mainly include unemployed, retired, or other occupations

**Table 2:** Impact of interventions on infectious disease-related conditions in children and adolescents

Indicators	Time	Boy			Girls			Total	
		Intervention group	Control group		Intervention group	Control group		Intervention group	Control group
***Individual level***									
Wash your hands before meals (yes)	Baseline(%)	46.51	49.02		46.90	48.86		46.70	48.94
	Post-intervention(%)	47.77	49.04		48.96	49.35		48.36	49.19
	Change (%)	1.26	0.02		2.06	0.49		1.66	0.25
	Effect for OR	0.95(0.89–1.02)			0.94(0.87–1.01)			**0.94(0.90–0.99)**	
	*p*-values	0.159			0.073			**0.024**	
Wash your hands when you go home (yes)	Baseline(%)	69.56	70.08		73.69	72.30		71.59	71.17
	Post-intervention(%)	73.49	72.29		76.98	75.08		75.21	73.66
	Change (%)	3.93	2.21		3.29	2.78		3.62	2.49
	Effect for OR	**0.92(0.85–0.99)**			0.97(0.89–1.05)			**0.94(0.89–0.99)**	
	*p*-values	**0.028**			0.414			**0.032**	
***Interpersonal level***									
No sharing of towels or bedding with others (yes)	Baseline(%)	64.95	62.38		68.25	66.44		66.60	64.40
	Post-intervention(%)	63.23	61.57		66.31	66.92		64.75	64.23
	Change (%)	-1.72	-0.81		-1.94	0.48		-1.85	-0.17
	Effect for OR	1.04(0.92–1.19)			1.12(0.98–1.27)			1.08(0.98–1.18)	
	*p*-values	0.514			0.104			1.113	
Classmates around you who are sick or not fully recovered still come to school (yes)	Baseline(%)	71.87	70.47		75.11	74.54		73.49	72.51
	Post-intervention(%)	71.81	71.38		74.09	74.86		72.95	73.12
	Change (%)	-0.06	0.91		-1.02	0.32		-0.54	0.61
	Effect for OR	1.05(0.95–1.16)			1.08 (0.977-1.20)			1.06(0.99–1.15)	
	*p*-values	0.359			0.152			0.096	
***Organization level***									
Acquire knowledge about infectious diseases mainly from course studies and lectures (yes)	Baseline(%)	67.09	65.99		68.56	68.11		67.81	67.04
	Post-intervention(%)	72.81	69.35		73.48	72.03		73.14	70.67
	Change (%)	5.72	3.36		4.92	3.92		5.33	3.63
	Effect for OR	**0.89(0.82–0.96)**			0.95(0.88–1.03)			**0.92(0.87–0.97)**	
	*p*-values	**0.003**			0.192			**0.002**	
Acquire knowledge about infectious diseases mainly from teachers (yes)	Baseline(%)	69.71	70.96		72.61	74.91		71.14	72.92
	Post-intervention(%)	74.56	73.25		79.11	79.12		76.82	76.17
	Change (%)	4.85	2.29		6.50	4.21		5.68	3.25
	Effect for OR	**0.88(0.81–0.96)**			**0.89(0.82–0.97)**			**0.86(0.83–0.94)**	
	*p*-values	**0.002**			**0.008**			**0.001**	
***Community level***									
When you want to spit in a public place, you spit on a tissue or handkerchief (yes)	Baseline(%)	49.77	51.54		67.36	67.20		58.43	59.26
	Post-intervention(%)	54.81	54.78		72.29	71.64		63.42	63.09
	Change (%)	5.04	3.24		4.93	4.44		4.99	3.83
	Effect for OR	**0.93(0.87-1.00)**			0.98(0.90–1.05)			0.95(0.91-1.00)	
	*p*-values	**0.042**			0.543			0.064	
Cover your cough or sneeze with a tissue or handkerchief in public places (yes)	Baseline(%)	31.52	32.13		34.48	34.75		32.98	33.43
	Post-intervention(%)	34.01	33.56		38.58	36.72		36.26	35.12
	Change (%)	2.49	1.43		4.10	1.97		3.28	1.69
	Effect for OR	0.95(0.88–1.03)			**0.91(0.85–0.98)**			**0.93(0.88–0.98)**	
	*p*-values	0.198			**0.011**			**0.008**	
***Policy level***									
If you were sick, you will told the school teacher (yes)	Baseline(%)	38.87	41.31		35.87	37.65		37.40	39.51
	Post-intervention(%)	39.21	40.70		36.12	37.47		37.69	39.11
	Change (%)	0.34	-0.61		0.25	-0.18		0.29	-0.40
	Effect for OR	0.96(0.89–1.03)			0.98(0.90–1.06)			0.97(0.92–1.02)	
	*p*-values	0.264			0.558			0.225	
When resuming classes due to illness, submit a class resumption certificate to the teacher (yes)	Baseline(%)	34.95	35.31		33.61	33.42		34.29	34.38
	Post-intervention(%)	37.80	38.21		36.82	37.36		37.32	37.79
	Change (%)	2.85	2.90		3.21	3.94		3.03	3.41
	Effect for OR	1.00(0.93–1.08)			1.03(0.95–1.12)			1.02(0.96–1.07)	
	*p*-values	0.946			0.416			0.536	

Note: The model was adjusted for age, province, and urban/rural area

## Discussion

After a six-month health education intervention on infectious diseases, our study found that the health behaviors related to infectious diseases among children and adolescents in the intervention group improved significantly, particularly among girls and younger age groups, compared to the control group. The intervention measures provided opportunities for children and adolescents to acquire knowledge about infectious diseases through courses, lectures, teachers, and doctors. These findings support the effectiveness of school-based interventions in preventing and controlling childhood infectious diseases in the post-COVID-19 epidemic era. Our study provides valuable evidence for comprehensive intervention strategies against infectious diseases in the childhood population.

Comprehensive intervention strategies, including strengthening healthy lifestyles, health education on infectious diseases, self-monitoring management, and school support, were found to be effective in controlling the spread of infectious diseases and reducing the global public health burden. This study confirmed the practicality of a multi-dimensional health intervention strategy based on the social-ecological model. Although the intervention period was only six months, the level of health education on infectious diseases in the intervention group was higher than that in the control group at the individual and community levels, particularly in hand hygiene and cough etiquette [21]. Besides, Polish scholars showed that SARS-CoV-2 / COVID-19 health education interventions were effective in reducing the basic knowledge gap and raising the awareness of high school students [22]. However, the health behavior of children and adolescents had not changed much at the interpersonal level, indicating the need for longer interventions, which was different from previous studies [23, 24].

Our study showed that health interventions were more effective in rural areas compared to urban areas, at various levels including individual, interpersonal, community, and organizational levels. This could have been due to better adherence to the interventions in rural areas. The success of interventions targeting children’s health behavior change and increased health knowledge may have depended on their acceptance of the intervention. Health education played a crucial role in raising awareness about potential health effects, as evident in studies conducted in Malaysia [25] and other low- and middle-income countries [26] where health education interventions were identified as highly effective in promoting personal hygiene and health literacy among students in rural areas. However, it’s important to acknowledge that barriers to implementation at the district level could have impacted the effectiveness of interventions. Factors such as economic status of the district and willingness to comply with instructions [27–29] should have been considered as potential barriers to intervention implementation. Proper consideration of these factors was essential in ensuring successful implementation of interventions and achieving desired outcomes.

Previous studies have mostly focused on a single dimension of infectious disease intervention strategies, but this study shows that a multidimensional health intervention strategy based on the social-ecological model can effectively control the spread of infectious diseases and reduce the global public health burden [30]. A COVID-19 study in China [31] showed that females had a significantly higher score in health education in terms of the “main clinical manifestation of COVID-19”, in line with the result of an investigation on Middle East Respiratory Syndrome in Saudi Arabian [32]. At the individual level, the intervention improved the health behaviors of infectious diseases, especially among girls, who may have broader access to health education [33]. In this context, factors of broader access to health education might have influenced the association between girls and boys. Another possible behavioral explanation is that the acquisition of broader health education knowledge may reflect higher health awareness and behaviors [22]. These findings aligned with previous studies that showed that boys were less likely than girls to take preventive and protective measures in response to infectious diseases like SARS and MERS [34–36]. These results highlighted the importance of including health education interventions targeted at boys in future intervention plans. For instance, sending health information to women who lived with boys, such as sisters or mothers, impacted the boys’ practices, as evidenced by a study conducted in Hong Kong [35]. Therefore, it is important to increase the accessibility of health education for children and adolescents, especially among boys. Additionally, older children and adolescents tend to have a higher understanding and acceptance of health education on infectious diseases, while younger children may need more attention. The potential impact of health education on infectious diseases for schools and preschool children should also be considered.

School-related support measures were critical to helping children and adolescents maintain healthy lifestyles, since they offer health education and a healthy environment, and have a powerful social network of teachers and peers [37]. A Nigerian study of the impact of adjusting school curricula on infectious diseases showed that, through the realignment of the Nigerian secondary school curriculum, students could be better re-positioned in the fight against communicable diseases [38]. At the organizational level, the present intervention measures effectively increase the way children and adolescents acquire health education on infectious diseases, such as getting information from teachers and doctors. Similarly, the lack of school engagement in increasing students’ awareness regarding different aspects of COVID-19 resulted in students acquiring less knowledge of COVID-19 from schools [39]. It was worth mentioning that health education on infectious diseases could be obtained through online classes during the COVID-19 lockdown period [40]. Mass media were valuable resources for efficiently forming people’s knowledge, improving their risk perception, and adequately informing people about risks and precautions [40]. Therefore, schools should be required to implement practical and effective methods to increase students’ access to knowledge and to deliver specific health information to students directly.

Our study had the advantage of a nationally representative sample from seven provinces in China, and we focus on children and adolescents aged 6–18 years old, covering a wide age range. However, some limitations should be paid attention to when interpreting the findings. First, because we excluded children and adolescents who dropped out, we were unable to comment on interventions for infectious diseases in that drop-out. Second, this study was based on a questionnaire study, and one of the limitations of the questionnaire study may be that it cannot explain the root cause of the results. Another limitation of questionnaire-based research may be due to the likely tendency of respondents to comply, which may favor positive outcomes to some extent. However, in this process, we carried out strict quality control to ensure reliability. In terms of infectious disease information, parent-child questionnaires for children in grades 1–3 were all reported by parents. In addition, trained project members explained all questionnaires in detail. These project members were given appropriate guidance as effectively as possible. For the same participants, the questionnaire was reviewed in 3% within a week [41]. As a result, the quality of infectious disease-related information was largely assured. Third, questionnaires based on children’s and adolescents’ recollections may not be representative of the long-term situation. Further research with more information related to infectious diseases is needed in the future. Fourth, the level of parental involvement in health education may have varied depending on the source of intervention information, particularly when the information was provided by teachers or school doctors. In China, parents may have placed a high level of trust in school teachers and prioritized their advice due to cultural norms where school-age children typically followed their parents’ directions. Therefore, it was important to train and incentivize health education teachers and school doctors in China. However, it is important to note that this intervention may not have been applicable in other countries around the world due to cultural differences.

## Conclusion

Our study has demonstrated that implementing school-based health education programs focused on infectious disease knowledge intervention can effectively improve childhood health behaviors related to infectious diseases at the individual, organizational, and community levels within China’s multi-center environment. Given the global COVID-19 pandemic, providing active health education on infectious diseases to children and adolescents could be an effective strategy for controlling disease transmission, thus highlighting the significant public health implications of such interventions, which should be initiated during early childhood.

## Electronic supplementary material

Below is the link to the electronic supplementary material.

Supplementary Material 1

## Data Availability

The datasets generated and/or analysed during the current study are not publicly available due confidentiality provisions in the consent process, but are available from the corresponding author on reasonable request.

## Abbreviations

COVID-19: Corona Virus Disease 2019
HLI-CCA: Chinese Children and Adolescents Healthy Lifestyle Intervention
SEM: Social ecological model
