# Supplementary material for: Enhancing the effectiveness of infectious disease health education for children and adolescents in China: a national multicenter school-based trial
Source: BMC Public Health. 2023 Jun 15;23:1161. doi: 10.1186/s12889-023-16000-3 (PMC10273566; doi:10.1186/s12889-023-16000-3)
Supplement: Supplementary file 1 — Supplementary Material 1 [file 12889_2023_16000_MOESM1_ESM.docx]

Supplementary Material

Supplementary Table 1. Baseline characteristics between intervention and control groups of the study population.

| characteristics | Primary School | |  | Junior High School | |  | High School | | t/χ^2^ | *p-*value |
| --- | --- | --- | --- | --- | --- | --- | --- | --- | --- | --- |
|  | Intervention group | Control group |  | Intervention group | Control group |  | Intervention group | Control group |  |  |
| N | 14007 | 14116 |  | 7001 | 5459 |  | 5583 | 4752 | -2.364 | 0.018 |
| Boys (%) | 7436(53.09) | 7428(52.62) |  | 3550(50.71) | 2777(50.87) |  | 2511(44.98) | 21459(45.14) | -0.020 | 0.984 |
| Age(years) | 8.19(1.45) | 8.22(1.45) |  | 12.62(0.77) | 12.65(0.78) |  | 15.64(0.81) | 15.69(0.89) | -8.510 | ＜0.001 |
| City(%) | 8871(63.33) | 8346(59.12) |  | 3931(56.15) | 3675(67.32) |  | 3220(57.68) | 2886(60.73) | -2.364 | 0.018 |
| Primary caregivers, n (%) |  |  |  |  |  |  |  |  |  |  |
| Parents | 12609(92.46) | 12931(93.75) |  | 6057(90.32) | 4851(91.53) |  | 4371(79.85) | 3452(74.64) | -1.060 | 0.287 |
| Socio-demographics, n (%) | | | | | | | | | | |
| Only child | 9638(68.81) | 9683(68.60) |  | 4929(70.40) | 3487(63.88) |  | 3806(68.17) | 3260(68.60) | 3.773 | ＜0.001 |
| Father’s educational level |  |  |  |  |  |  |  |  |  |  |
| Junior high school and below | 4671(35.11) | 5680(42.11) |  | 2871(47.80) | 2718(53.61) |  | 2225(46.22) | 2059(56.83) | -17.312 | ＜0.001 |
| High School and Junior College | 3792(28.50) | 3858(28.60) |  | 1406(23.41) | 1357(26.77) |  | 1448(30.08) | 926(25.56) |  |  |
| College and above | 4842(36.39) | 3952(29.30) |  | 1729(28.79) | 995(19.63) |  | 1141(23.70) | 638(17.61) |  |  |
| Mather’s educational level |  |  |  |  |  |  |  |  |  |  |
| Junior high school and below | 5221(39.34) | 6133(45.69) |  | 3029(50.40) | 2945(58.19) |  | 2447(50.81) | 2282(62.73) | -16.245 | ＜0.001 |
| High School and Junior College | 3675(27.69) | 3601(26.83) |  | 1401(23.31) | 1231(24.32) |  | 1381(28.68) | 791(21.74) |  |  |
| College and above | 4375(32.97) | 3688(27.48) |  | 1580(26.29) | 885(17.49) |  | 988(20.51) | 565(15.53) |  |  |
| Father occupation |  |  |  |  |  |  |  |  |  |  |
| Administrator and clerk | 859(7.91) | 719(6.66) |  | 367(7.79) | 242(6.35) |  | 388(10.22) | 157(5.39) | 9.980 | ＜0.001 |
| Professional and technical | 1644(15.13) | 1626(15.07) |  | 777(16.49) | 509(13.36) |  | 537(14.14) | 340(11.68) |  |  |
| Commerce and services | 4178(38.46) | 3796(35.17) |  | 1367(29.01) | 1233(32.36) |  | 1128(29.70) | 733(25.18) |  |  |
| Other | 4182(38.50) | 4651(43.10) |  | 2201(46.71) | 1826(47.93) |  | 1745(45.95) | 1681(57.75) |  |  |
| Mother occupation |  |  |  |  |  |  |  |  |  |  |
| Administrator and clerk | 407(3.56) | 303(2.64) |  | 178(3.50) | 102(2.47) |  | 187(4.60) | 80(2.54) | 9.600 | ＜0.001 |
| Professional and technical | 1486(13.00) | 1448(12.62) |  | 707(13.91) | 413(10.01) |  | 499(12.27) | 296(9.38) |  |  |
| Commerce and services | 4562(39.90) | 4188(36.50) |  | 1538(30.27) | 1413(34.24) |  | 1175(28.89) | 811(25.71) |  |  |
| Other | 4978(43.54) | 5536(48.24) |  | 2658(52.31) | 2199(53.28) |  | 2206(54.24) | 1967(62.37) |  |  |
| Monthly household income (RMB) |  |  |  |  |  |  |  |  |  |  |
| < 2000 | 919(15.26) | 1035(15.32) |  | 665(19.48) | 611(20.64) |  | 447(16.98) | 481(24.07) | -2.572 | 0.006 |
| 2,000–5,000 | 2851(47.34) | 3198(47.33) |  | 1715(50.23) | 1522(51.42) |  | 1316(50.00) | 970(48.55) |  |  |
| 5,000-8,000 | 2253(37.41) | 2524(37.35) |  | 1034(30.29) | 827(27.94) |  | 869(33.02) | 547(27.38) |  |  |

Note: Other occupations of father and mother mainly include unemployed, retired, or other occupations

Supplementary Table 2. Impact of interventions on infectious disease-related conditions among children and adolescents (by urban/rural area).

| Indicators | Time | urban | |  | rural | |  | Total | |
| --- | --- | --- | --- | --- | --- | --- | --- | --- | --- |
|  |  | Intervention group | Control group |  | Intervention group | Control group |  | Intervention group | Control group |
|  | | | | | | | | | |
| Wash your hands before meals (yes) | Baseline(%) | 46.29 | 46.19 |  | 47.34 | 53.31 |  | 46.70 | 48.94 |
|  | Post-intervention(%) | 47.94 | 48.30 |  | 48.98 | 50.61 |  | 48.36 | 49.19 |
|  | Change (%) | 1.65 | 2.11 |  | 1.64 | -2.70 |  | 1.66 | 0.25 |
|  | Effect for OR | 1.02(0.96-1.09) | |  | **0.83(0.77-0.90)** | |  | **0.94(0.90-0.99)** | |
|  | *p*-values | 0.576 | |  | **0.000** | |  | **0.024** | |
| Wash your hands when you go home (yes) | Baseline(%) | 73.72 | 71.37 |  | 68.37 | 70.87 |  | 71.59 | 71.17 |
|  | Post-intervention(%) | 76.59 | 75.01 |  | 73.11 | 71.53 |  | 75.21 | 73.66 |
|  | Change (%) | 2.87 | 3.64 |  | 4.74 | 0.66 |  | 3.62 | 2.49 |
|  | Effect for OR | 1.03(0.96-1.11) | |  | **0.82(0.75-0.90)** | |  | **0.94(0.89-0.99)** | |
|  | *p*-values | 0.374 | |  | **0.001** | |  | **0.032** | |
| ***Interpersonal level*** | | | | | | | | | |
| No sharing of towels or bedding with others (yes) | Baseline(%) | 68.13 | 66.65 |  | 64.51 | 61.21 |  | 66.60 | 64.40 |
|  | Post-intervention(%) | 65.79 | 65.47 |  | 63.31 | 62.49 |  | 64.75 | 64.23 |
|  | Change (%) | -2.34 | -1.18 |  | -1.20 | 1.28 |  | -1.85 | -0.17 |
|  | Effect for OR | 1.055(0.93-1.18) | |  | 1.12(0.97-1.29) | |  | 1.08(0.98-1.18) | |
|  | *p*-values | 0.440 | |  | 0.110 | |  | 1.113 | |
| Classmates around you who are sick or not fully recovered still come to school (yes) | Baseline(%) | 52.26 | 52.92 |  | 54.19 | 53.69 |  | 53.04 | 53.22 |
|  | Post-intervention(%) | 52.20 | 51.48 |  | 52.32 | 56.35 |  | 52.25 | 53.37 |
|  | Change (%) | -0.06 | -1.44 |  | -1.87 | 2.66 |  | -0.79 | 0.15 |
|  | Effect for OR | 0.99（0.90-1.10） | |  | **1.18（1.05-1.32）** | |  | 1.06（0.99-1.14） | |
|  | *p*-values | 0.909 | |  | **0.004** | |  | 0.106 | |
| ***Organization level*** | | | | | | | | | |
| Acquire knowledge about infectious diseases mainly from course studies and lectures (yes) | Baseline(%) | 68.29 | 67.25 |  | 67.11 | 66.70 |  | 67.81 | 67.04 |
|  | Post-intervention(%) | 73.64 | 71.50 |  | 72.41 | 69.40 |  | 73.14 | 70.67 |
|  | Change (%) | 5.35 | 4.25 |  | 5.30 | 2.70 |  | 5.33 | 3.63 |
|  | Effect for OR | 0.94（0.88-1.01） | |  | **0.88（0.81-0.96）** | |  | **0.92（0.87-0.97）** | |
|  | *p*-values | 0.107 | |  | **0.005** | |  | **0.002** | |
| Acquire knowledge about infectious diseases mainly from teachers (yes) | Baseline(%) | 70.01 | 72.28 |  | 72.76 | 73.90 |  | 71.14 | 72.92 |
|  | Post-intervention(%) | 76.71 | 76.27 |  | 76.98 | 76.01 |  | 76.82 | 76.17 |
|  | Change (%) | 6.70 | 3.99 |  | 4.22 | 2.11 |  | 5.68 | 3.25 |
|  | Effect for OR | **0.88（0.81-0.94）** | |  | **0.89（0.81-0.98）** | |  | **0.89（0.83-0.94）** | |
|  | *p*-values | **0.001** | |  | **0.017** | |  | **＜0.001** | |
| ***Community level*** | | | | | | | | | |
| When you want to spit in a public place, you spit on a tissue or handkerchief (yes) | Baseline(%) | 57.52 | 56.96 |  | 59.81 | 62.91 |  | 58.43 | 59.26 |
|  | Post-intervention(%) | 62.64 | 61.26 |  | 64.59 | 65.99 |  | 63.42 | 63.09 |
|  | Change (%) | 5.12 | 4.30 |  | 4.78 | 3.08 |  | 4.99 | 3.83 |
|  | Effect for OR | 0.96（0.90-1.03） | |  | 0.93（0.86-1.01） | |  | 0.95（0.90-1.00） | |
|  | *p*-values | 0.282 | |  | 0.093 | |  | 0.061 | |
| Cover your cough or sneeze with a tissue or handkerchief in public places (yes) | Baseline(%) | 30.74 | 30.40 |  | 36.37 | 38.22 |  | 32.98 | 33.43 |
|  | Post-intervention(%) | 33.77 | 32.46 |  | 40.01 | 39.31 |  | 36.26 | 35.12 |
|  | Change (%) | 3.03 | 2.06 |  | 3.64 | 1.09 |  | 3.28 | 1.69 |
|  | Effect for OR | 0.96（0.89-1.03） | |  | **0.90（0.83-0.97）** | |  | **0.93（0.89-0.98）** | |
|  | *p*-values | 0.215 | |  | **0.008** | |  | **0.008** | |
| ***Policy level*** | | | | | | | | | |
| If you were sick, you will told the school teacher (yes) | Baseline(%) | 36.98 | 38.15 |  | 38.02 | 41.66 |  | 37.40 | 39.51 |
|  | Post-intervention(%) | 37.76 | 38.75 |  | 37.57 | 39.67 |  | 37.69 | 39.11 |
|  | Change (%) | 0.78 | 0.60 |  | -0.45 | -1.99 |  | 0.29 | -0.40 |
|  | Effect for OR | 0.99(0.93-1.06) | |  | 0.93(0.85-1.01) | |  | 0.97(0.92-1.02) | |
|  | *p*-values | 0.811 | |  | 0.090 | |  | 0.227 | |
| When resuming classes due to illness, submit a class resumption certificate to the teacher (yes) | Baseline(%) | 33.20 | 34.96 |  | 35.73 | 33.59 |  | 34.29 | 34.38 |
|  | Post-intervention(%) | 37.18 | 39.79 |  | 37.50 | 35.11 |  | 37.32 | 37.79 |
|  | Change (%) | 3.98 | 4.83 |  | 1.77 | 1.52 |  | 3.03 | 3.41 |
|  | Effect for OR | 1.03（0.96-1.11） | |  | 0.99（0.91-1.08） | |  | 1.02（0.96-1.08） | |
|  | *p*-values | 0.396 | |  | 0.784 | |  | 0.538 | |

Note: Model was adjusted for age, sex, province.

Supplementary Table 3. Impact of interventions on infectious disease-related conditions among children and adolescents (by age).

| Indicators | Time | 6-8(years) | |  | 9-11(years) | |  | 12-14(years) | |  | 15-18(years) | |  | Total | |
| --- | --- | --- | --- | --- | --- | --- | --- | --- | --- | --- | --- | --- | --- | --- | --- |
|  |  | Intervention group | Control group |  | Intervention group | Control group |  | Intervention group | Control group |  | Intervention group | Control group |  | Intervention group | Control group |
| Individual level | | | | | | | | | | | | | | | |
| Wash your hands before meals (yes) | Baseline | 51.16 | 54.84 |  | 55.53 | 59.64 |  | 44.8 | 43.89 |  | 32.27 | 29.62 |  | 46.7 | 48.94 |
|  | Post-intervention | 52.1 | 54.15 |  | 54.87 | 59.78 |  | 46.87 | 43.21 |  | 37.13 | 32.74 |  | 48.36 | 49.19 |
|  | Change | 0.94 | -0.69 |  | -0.66 | 0.14 |  | 2.07 | -0.68 |  | 4.86 | 3.12 |  | 1.66 | 0.25 |
|  | Effect for OR | 0.94(0.86-1.02) | |  | 1.03(0.93-1.14) | |  | **0.89(0.81-0.99)** | |  | 0.93(0.83-1.05) | |  | **0.95(0.90-0.99)** | |
|  | p-values | 0.147 | |  | 0.522 | |  | **0.031** | |  | 0.260 |  |  | **0.025** | |
| Wash your hands when you go home (yes) | Baseline | 69.57 | 72.89 |  | 75.38 | 75.7 |  | 73.05 | 70.45 |  | 68.27 | 65.91 |  | 71.59 | 71.17 |
|  | Post-intervention | 72.9 | 1.89 |  | 78.27 | 78.16 |  | 76.72 | 72.38 |  | 73.08 | 70.11 |  | 75.21 | 73.66 |
|  | Change | 3.33 |  |  | 2.89 | 2.46 |  | 3.67 | 1.93 |  | 4.81 | 4.2 |  | 3.62 | 2.49 |
|  | Effect for OR | 0.93(0.85-1.03) | |  | 0.98(0.87-1.10) | |  | 0.90(0.81-1.01) | |  | 0.96(0.85-1.09) | |  | **0.94(0.89-0.99)** | |
|  | p-values | 0.170 | |  | 0.694 | |  | 0.081 | |  | 0.523 | |  | **0.032** | |
| Interpersonal level | | | | | | | | | | | | | | | |
| No sharing of towels or bedding with others (yes) | Baseline | 59.72 | 55.92 |  | 63.75 | 63.18 |  | 70.12 | 68.79 |  | 72.78 | 71.98 |  | 66.6 | 64.4 |
|  | Post-intervention | 60.15 | 59.97 |  | 61.47 | 60.3 |  | 68.11 | 65.89 |  | 68.13 | 70.34 |  | 64.75 | 64.23 |
|  | Change | 0.43 | 4.05 |  | -2.28 | -2.88 |  | -2.01 | -2.9 |  | -4.65 | -1.64 |  | -1.85 | -0.17 |
|  | Effect for OR | 1.16(0.98-1.37) | |  | 0.98(0.80-1.21) | |  | 0.97(0.81-1.16) | |  | 1.15(0.95-1.39) | |  | 1.08(0.98-1.18) | |
|  | p-values | 0.073 | |  | 0.859 | |  | 0.749 | |  | 0.140 | |  | 0.107 | |
| Classmates around you who are sick or not fully recovered still come to school (yes) | Baseline | 68.58 | 67.22 |  | 66.51 | 67.14 |  | 73.2 | 71.66 |  | 86.87 | 87.5 |  | 73.49 | 72.51 |
|  | Post-intervention | 68.59 | 69.75 |  | 67.53 | 68.93 |  | 73.51 | 72.99 |  | 83.9 | 83.88 |  | 72.95 | 73.12 |
|  | Change | 0.01 | 2.53 |  | 1.02 | 1.79 |  | 0.31 | 1.33 |  | -2.97 | -3.62 |  | -0.54 | 0.61 |
|  | Effect for OR | 1.14(0.99-1.30) | |  | 1.04(0.91-1.18) | |  | 1.05(0.91-1.221) | |  | 0.94(0.77-1.15) | |  | 1.06(0.99-1.14) | |
|  | p-values | 0.064 | |  | 0.608 | |  | 0.520 | |  | 0.552 | |  | 0.269 | |
| Organization level | | | | | | | | | | | | | | | |
| Acquire knowledge about infectious diseases mainly from classrooms and lectures (yes) | Baseline | 63.36 | 63.91 |  | 67.24 | 65.01 |  | 71.68 | 72.87 |  | 70 | 67.84 |  | 67.81 | 67.04 |
|  | Post-intervention | 68.55 | 67.5 |  | 73.71 | 68.8 |  | 77.96 | 76.19 |  | 72.8 | 71.87 |  | 73.14 | 70.67 |
|  | Change | 5.19 | 3.59 |  | 6.47 | 3.79 |  | 6.28 | 3.32 |  | 2.8 | 4.03 |  | 5.33 | 3.63 |
|  | Effect for OR | 0.92(0.84-1.02） | |  | **0.87(0.78-0.97)** | |  | **0.85(0.76-0.96）** | |  | 1.06(0.93-1.20) | |  | **0.92(0.87-0.97)** | |
|  | p-values | 0.116 | |  | **0.012** | |  | **0.008** | |  | 0.393 | |  | **0.002** |  |
| Acquire knowledge about infectious diseases mainly from teachers (yes) | Baseline | 71.4 | 73.06 |  | 67.04 | 68.69 |  | 71.48 | 79.49 |  | 75.38 | 70.65 |  | 71.14 | 72.92 |
|  | Post-intervention | 72.9 | 72.97 |  | 74.14 | 72.85 |  | 83.36 | 82.85 |  | 77.17 | 78.13 |  | 76.82 | 76.17 |
|  | Change | 1.5 | -0.09 |  | 7.1 | 4.16 |  | 11.88 | 3.36 |  | 1.79 | 7.48 |  | 5.68 | 3.25 |
|  | Effect for OR | 0.92(0.83-1.03) | |  | **0.87(0.78-0.97)** | |  | **0.62(0.55-0.71)** | |  | 1.34(1.17-1.54) | |  | **0.88(0.83-0.94)** | |
|  | p-values | 0.138 | |  | **0.013** | |  | **<0.001** | |  | <0.001 | |  | **<0.001** | |
| Community level | | | | | | | | | | | | | | | |
| When you want to spit in a public place, you spit on a tissue or handkerchief (yes) | Baseline | 49.91 | 52.26 |  | 62.79 | 64.6 |  | 61.89 | 61.77 |  | 61.37 | 60.54 |  | 58.43 | 59.26 |
|  | Post-intervention | 57.79 | 56.71 |  | 68.24 | 69.26 |  | 64.45 | 63.59 |  | 64.72 | 64.62 |  | 63.42 | 63.09 |
|  | Change | 7.88 | 4.45 |  | 5.45 | 4.66 |  | 2.56 | 1.82 |  | 3.35 | 4.08 |  | 4.99 | 3.83 |
|  | Effect for OR | **0.87(0.79-0.95)** | |  | 0.97(0.87-1.08) | |  | 0.97(0.87-1.08) | |  | 1.03(0.92-1.17) | |  | 0.95(0.90-1.00) | |
|  | p-values | **0.002** | |  | 0.549 | |  | 0.544 | |  | 0.589 | |  | 0.059 | |
| Cover your cough or sneeze with a tissue or handkerchief in public places (yes) | Baseline | 30.73 | 33.11 |  | 40.31 | 38.62 |  | 34.77 | 34.25 |  | 25.44 | 25.57 |  | 32.98 | 33.43 |
|  | Post-intervention | 34.34 | 34.76 |  | 43.61 | 41.26 |  | 36.27 | 34.44 |  | 30.5 | 27.74 |  | 36.26 | 35.12 |
|  | Change | 3.61 | 1.65 |  | 3.3 | 2.64 |  | 1.5 | 0.19 |  | 5.06 | 2.17 |  | 3.28 | 1.69 |
|  | Effect for OR | 0.91(0.83-1.00) | |  | 0.97(0.88-1.08) | |  | 0.95(0.85-1.05) | |  | **0.87(0.76-0.98)** | |  | **0.93(0.88-0.98)** | |
|  | p-values | 0.055 | |  | 0.618 | |  | 0.295 | |  | **0.028** | |  | **0.008** | |
| Policy level | | | | | | | | | | | | | | | |
| If you were sick, you will told the school teacher (yes) | Baseline | 51.67 | 54.86 |  | 49.28 | 51.79 |  | 29.33 | 27.24 |  | 12.91 | 10.47 |  | 37.4 | 39.51 |
|  | Post-intervention | 53.03 | 54.44 |  | 49.03 | 50.71 |  | 28.01 | 26.37 |  | 14.33 | 11.58 |  | 37.69 | 39.11 |
|  | Change | 1.36 | -0.42 |  | -0.25 | -1.08 |  | -1.32 | -0.87 |  | 1.42 | 1.11 |  | 0.29 | -0.4 |
|  | Effect for OR | 0.93(0.85-1.02) | |  | 0.97(0.88-1.07) | |  | 1.02(0.91-1.14) | |  | 0.99(0.83-1.18) | |  | 0.97(0.92-1.02) | |
|  | p-values | 0.115 | |  | 0.508 | |  | 0.724 | |  | 0.934 | |  | 0.269 | |
| When resuming classes due to illness, submit a class resumption certificate to the teacher (yes) | Baseline | 36.91 | 37.94 |  | 32.67 | 33.7 |  | 35.35 | 34.51 |  | 31.36 | 29.58 |  | 34.29 | 34.38 |
|  | Post-intervention | 36.31 | 40.16 |  | 35.08 | 36.32 |  | 40.46 | 37.9 |  | 37.08 | 35.92 |  | 37.32 | 37.79 |
|  | Change | -0.6 | -0.6 |  | 2.41 | 2.62 |  | 5.11 | 5.11 |  | 3.39 | 5.72 |  | 6.34 | 3.03 |
|  | Effect for OR | 1.13(1.02-1.25) | |  | 1.01(0.90-1.12) | |  | 0.93(0.83-1.04) | |  | 1.04(0.92-1.18) | |  | 1.02（0.96-1.08） | |
|  | p-values | 0.020 | |  | 0.909 | |  | 0.209 | |  | 0.552 |  |  | 0.541 | |

Note: Model was adjusted for sex, province, and urban/rural area.

Supplementary Table 4. Impact of interventions on infectious disease-related conditions among children and adolescents (by school level).

| Indicators | Time | Junior high school and below | |  | High School and Junior College | |  | College and above | |  | Total | |
| --- | --- | --- | --- | --- | --- | --- | --- | --- | --- | --- | --- | --- |
|  |  | Intervention group | Control group |  | Intervention group | Control group |  | Intervention group | Control group |  | Intervention group | Control group |
| Individual level | | | | | | | | | | | | |
| Wash your hands before meals (yes) | Baseline | 46.72 | 49.97 |  | 46.73 | 51.17 |  | 46.98 | 48.03 |  | 46.7 | 48.94 |
|  | Post-intervention | 48.52 | 49.62 |  | 48.48 | 50.47 |  | 48.49 | 49.31 |  | 48.36 | 49.19 |
|  | Change | 1.8 | -0.35 |  | 1.75 | -0.7 |  | 1.51 | 1.28 |  | 1.66 | 0.25 |
|  | Effect for OR | **0.91(0.84-1.00)** | |  | **0.90(0.82-1.00)** | |  | 0.99(0.91-1.08) | |  | **0.94(0.90-0.99)** | |
|  | p-values | **0.045** | |  | **0.043** | |  | 0.838 | |  | **0.024** | |
| Wash your hands when you go home (yes) | Baseline | 66.47 | 68.61 |  | 71.39 | 71.8 |  | 76.41 | 75.03 |  | 71.59 | 71.17 |
|  | Post-intervention | 71.34 | 70.52 |  | 75.09 | 74.45 |  | 79.45 | 78.11 |  | 75.21 | 73.66 |
|  | Change | 4.87 | 1.91 |  | 3.7 | 2.65 |  | 3.04 | 3.08 |  | 3.62 | 2.49 |
|  | Effect for OR | **0.87(0.79-0.96)** | |  | 0.95(0.85-1.06) | |  | 1.00(0.90-1.11) | |  | **0.94(0.89-0.99)** | |
|  | p-values | **0.003** | |  | 0.336 | |  | 0.931 | |  | **0.032** | |
| Interpersonal level | | | | | | | | | | | | |
| No sharing of towels or bedding with others (yes) | Baseline | 64.47 | 62.48 |  | 66 | 64.93 |  | 70.52 | 65.92 |  | 66.6 | 64.4 |
|  | Post-intervention | 63.06 | 62.44 |  | 65.63 | 62.03 |  | 66.55 | 68.44 |  | 64.75 | 64.23 |
|  | Change | -1.41 | -0.04 |  | -0.37 | -2.9 |  | -3.97 | 2.52 |  | -1.85 | -0.17 |
|  | Effect for OR | 1.07(0.93-1.24) | |  | 0.89(0.75-1.07) | |  | 1.34(1.11-1.61) | |  | 1.08(0.98-1.18) | |
|  | p-values | 0.349 | |  | 0.227 | |  | 0.002 | |  | 0.113 | |
| Classmates around you who are sick or not fully recovered still come to school (yes) | Baseline | 71.72 | 70.85 |  | 74.04 | 71.87 |  | 73.41 | 72.15 |  | 73.49 | 72.51 |
|  | Post-intervention | 70.46 | 71.74 |  | 73.96 | 72.48 |  | 73.11 | 73.08 |  | 72.95 | 73.12 |
|  | Change | -1.26 | 0.89 |  | -0.08 | 0.61 |  | -0.3 | 0.93 |  | -0.54 | 0.61 |
|  | Effect for OR | 1.12(0.99-1.26) | |  | 1.03(0.89-1.19) | |  | 1.07(0.94-1.23) | |  | 1.06(0.99-1.14) | |
|  | p-values | 0.079 | |  | 0.685 | |  | 0.281 | |  | 0.096 | |
| Organization level | | | | | | | | | | | | |
| Acquire knowledge about infectious diseases mainly from classrooms and lectures (yes) | Baseline | 65.34 | 65.43 |  | 67.93 | 68.02 |  | 70.18 | 68.94 |  | 67.81 | 67.04 |
|  | Post-intervention | 71.18 | 68.78 |  | 72.48 | 71.67 |  | 75.82 | 72.72 |  | 73.14 | 70.67 |
|  | Change | 5.84 | 3.35 |  | 4.55 | 3.65 |  | 5.64 | 3.78 |  | 5.33 | 3.63 |
|  | Effect for OR | **0.89(0.81-0.98）** | |  | 0.96(0.86-1.07) | |  | **0.90(0.81-1.00）** | |  | **0.92(0.87-0.97)** | |
|  | p-values | **0.016** | |  | 0.433 | |  | **0.045** | |  | **0.002** | |
| Acquire knowledge about infectious diseases mainly from teachers (yes) | Baseline | 70.79 | 74.31 |  | 72.02 | 73.47 |  | 72.82 | 73.74 |  | 71.14 | 72.92 |
|  | Post-intervention | 77.96 | 77.24 |  | 75.6 | 76.14 |  | 76.7 | 74.57 |  | 76.82 | 76.17 |
|  | Change | 7.17 | 2.93 |  | 3.58 | 2.67 |  | 3.88 | 0.83 |  | 5.68 | 3.25 |
|  | Effect for OR | **0.81(0.73-0.89)** | |  | 0.96(0.86-1.08) | |  | **0.85(0.77-0.95)** | |  | **0.89(0.83-0.94)** | |
|  | p-values | **<0.001** | |  | 0.500 | |  | **0.003** | |  | **<0.001** | |
| Community level | | | | | | | | | | | | |
| When you want to spit in a public place, you spit on a tissue or handkerchief (yes) | Baseline | 57.88 | 59.67 |  | 56.83 | 59.65 |  | 59.88 | 58.97 |  | 58.43 | 59.26 |
|  | Post-intervention | 63.17 | 63.94 |  | 62.52 | 62.62 |  | 64.79 | 63.28 |  | 63.42 | 63.09 |
|  | Change | 5.29 | 4.27 |  | 5.69 | 2.97 |  | 4.91 | 4.31 |  | 4.99 | 3.83 |
|  | Effect for OR | 0.96(0.88-1.05) | |  | **0.89(0.81-0.99)** | |  | 0.97(0.89-1.07) | |  | 0.95(0.91-1.00) | |
|  | p-values | 0.358 | |  | **0.026** | |  | 0.574 | |  | 0.059 | |
| Cover your cough or sneeze with a tissue or handkerchief in public places (yes) | Baseline | 34.59 | 36.32 |  | 32.9 | 33.43 |  | 32.51 | 31.52 |  | 32.98 | 33.43 |
|  | Post-intervention | 37.52 | 37.1 |  | 36.19 | 36.08 |  | 36.56 | 33.14 |  | 36.26 | 35.12 |
|  | Change | 2.93 | 0.78 |  | 3.29 | 2.65 |  | 4.05 | 1.62 |  | 3.28 | 1.69 |
|  | Effect for OR | **0.91(0.83-0.99)** | |  | 0.97(0.88-1.08) | |  | 0.91(0.81-1.03) | |  | **0.93(0.88-0.98)** | |
|  | p-values | **0.035** | |  | 0.573 | |  | 0.144 | |  | **0.008** | |
| Policy level | | | | | | | | | | | | |
| If you were sick, you will told the school teacher (yes) | Baseline | 35.64 | 38.43 |  | 36.8 | 40.81 |  | 42.79 | 44.76 |  | 37.4 | 39.51 |
|  | Post-intervention | 34.2 | 36.03 |  | 37.81 | 40.34 |  | 44.35 | 46.93 |  | 37.69 | 39.11 |
|  | Change | -1.44 | -2.4 |  | 1.01 | -0.47 |  | 1.56 | 2.17 |  | 0.29 | -0.4 |
|  | Effect for OR | 0.95(0.86-1.05) | |  | 0.93(0.84-1.04) | |  | 1.02(0.93-1.12) | |  | 0.97(0.92-1.02) | |
|  | p-values | 0.305 | |  | 0.195 | |  | 0.617 | |  | 0.225 | |
| When resuming classes due to illness, submit a class resumption certificate to the teacher (yes) | Baseline | 34.5 | 32.71 |  | 34.52 | 34.7 |  | 35.13 | 37.89 |  | 34.29 | 34.38 |
|  | Post-intervention | 36.27 | 35.13 |  | 37.8 | 38.3 |  | 39.42 | 41.99 |  | 37.32 | 37.79 |
|  | Change | 1.77 | 2.42 |  | 3.28 | 3.60 |  | 4.29 | 4.10 |  | 3.03 | 3.41 |
|  | Effect for OR | 1.03(0.94-1.14) | |  | 1.01(0.91-1.13) | |  | 0.99(0.90-1.09) | |  | 1.02(0.96-1.08) | |
|  | p-values | 0.536 | |  | 0.808 | |  | 0.806 | |  | 0.536 | |

Note: Model was adjusted for sex, province, urban/rural area, and age.
